# The shortage of surgeons in Japan: Results of an online survey of qualified teaching hospitals that take part in the surgical training programs for board certification by the Japan Surgical Society

**DOI:** 10.1007/s00595-023-02697-7

**Published:** 2023-05-16

**Authors:** Hideki Takami, Yasuhiro Kodera, Hidetoshi Eguchi, Minoru Kitago, Kenta Murotani, Satoshi Hirano, Yuko Kitagawa, Norihiko Ikeda, Masaki Mori

**Affiliations:** 1 Department of Gastroenterological Surgery, Nagoya University Graduate School of Medicine, 65, Tsurumai-Cho, Showa-Ku, Nagoya, Aichi 466-8550 Japan; 2 Department of Gastroenterological Surgery, Osaka University Graduate School of Medicine, 2-2, Yamadaoka, Suita, Osaka 565-0871 Japan; 3 Department of Surgery, Keio University School of Medicine, 35 Shinanomachi, Shinjuku-Ku, Tokyo, 160-8582 Japan; 4 Biostatics Center, Kurume University, Kurume, 830-0011 Japan; 5 Department of Gastroenterological Surgery II, Faculty of Medicine, Hokkaido University, Kita-Ku, Kita 15, Nishi 7, Sapporo, Hokkaido 860-8556 Japan; 6 Department of Surgery, Tokyo Medical University, 6-7-1 Nishishinjuku, Shinjuku-Ku, Tokyo, 160-0023 Japan; 7 School of Medicine, Tokai University, 143, Shimokasuya, Isehara, Kanagawa 259-1193 Japan; 8 The Japan Surgical Society, Tokyo, Japan

**Keywords:** Board certification, Surgical training, Regional healthcare

## Abstract

**Purpose:**

A collapse in regional healthcare through the maldistribution of physicians has been a long-debated issue in Japan and amidst this situation, a new system of board certification was initiated. The Japan Surgical Society (JSS) conducted a nation-wide survey to grasp the current distribution of surgeons in Japan, and their roles.

**Methods:**

All 1976 JSS-certified teaching hospitals were invited to respond to a web-based questionnaire. The responses were analyzed to seek a solution to the current issues.

**Results:**

Responses to the questionnaire were received from 1335 hospitals. The surgical departments of medical universities serve as an internal labor market and were the source of surgeons for most hospitals. More than 50% of teaching hospitals throughout the country claimed a shortage of surgeons even in well-populated prefectures such as Tokyo and Osaka. Hospitals rely on surgeons to cover the deficits in medical oncology, anesthesiology, and emergency medicine. These additional responsibilities were identified as significant predictors of a shortage of surgeons.

**Conclusions:**

Surgeon shortage is a serious issue throughout Japan. Given the limited number of surgeons and surgical trainees, hospitals should make every effort to recruit specialists in the additional fields where surgeons are filling the gaps and allow surgeons to engage more in surgery.

**Supplementary Information:**

The online version contains supplementary material available at 10.1007/s00595-023-02697-7.

## Introduction

The Japanese Medical Specialty Board (JMSB) [1] was founded in 2014, and a new system to oversee the training program and board certification process for 19 fundamental specialty areas was introduced in 2018. Medical societies that had been responsible for the board certification of each fundamental specialty area collaborated with the JMSB to establish a new certification system. Accordingly, the Japan Surgical Society (JSS) established a training system in which each of the main training facilities that fulfilled certain criteria (see Methods) was invited to organize a training program involving at least one other hospital as a partner training facility. Partner training facilities are expected to fulfill another set of criteria that is not as demanding as the criteria for the main training facilities. By 2021, 237 training programs had been approved by the JSS, and subsequently by the JMSB. These surgical training programs are located throughout Japan and expected to recruit trainees who have completed 2 years of their initial residency program. Typically, the trainees undergo 3 years of training, during which they are expected to spend at least 6 months at the main training facility and at one of the partner training facilities, and to participate in more than 350 surgical procedures, along with other requirements for the JSS board certification [2]. The capacity for the recruitment of trainees into each training program is based on the patient resources and training resources of the program.

The maldistribution of health providers is a serious problem in Japan [3], with hospitals in rural areas being more inclined to suffer from a shortage of physicians. Moreover, it is widely recognized that the number of surgeons is alarmingly insufficient. Graduates of Japanese medical schools undergo a matching process to select a hospital for their 2-year residency program, following which they are able to choose their specialty. There is a perception that choosing to become a surgeon is associated with an “uncontrollable lifestyle” [4] which many modern medical school graduates would consider physically, emotionally, and/or spiritually incompatible with the vision they have for their life [5]. Consequently, the maldistribution of doctors in terms of geography and specialties has not been resolved, despite recent expansion in the capacity of medical schools. The shortage of surgeons will likely have detrimental effects on hospitals that strive to fulfill the working-hour regulations required by the physician’s work style reforms, which will be implemented in April, 2024 [6]. The Ministry of Health, Labor and Welfare has concerns about the feasibility of these work style reforms and has attempted to control the distribution of physicians through the new system of board certification, which started in 2018, 6 years prior to the work-style reforms. In line with the government’s policy, the JMSB established upper-limits in the number of trainees who can apply for a specialty in a given prefecture, and attempted to modify the rules of board certification to encourage the trainees and, ultimately, the board-certified doctors, to spend part of their training or career in rural areas in an attempt to remedy the maldistribution.

Although the JSS was offset by these political intents behind the new certification system, it will need to act positively to find ways to improve the current maldistribution of surgeons for the benefit of regional healthcare. Accordingly, the JSS conducted nation-wide surveillance to identify the hardship of surgeons across all districts and sent out a questionnaire to which representatives of all main and partner training facilities were asked to respond. This article summarizes the findings of this survey.

## Methods

### Questionnaires

To fully understand the current status and identify the causes of the shortage of surgeons at each teaching hospital, we prepared a questionnaire comprising 19 questions in collaboration with the Certification System Committee of the JSS (Table 1). The questionnaire was installed into a web-based questionnaire using Microsoft Forms and an e-mail inviting participation was sent to the representatives of all teaching hospitals approved as either a main or a partner training facility by the JMSB. The questionnaires were open for response between October 26, 2021 and November 12, 2021. The responses were collected and stored in the database at the JSS office to be analyzed by the authors.

**Table 1:** Questions asked in the Questionnaire

No	Questions	How a question is answered	
1	What is the name of your hospital?	Free writing	
2	In which prefecture is your hospital located?	Free writing	
3	What is the facility number of the hospital endowed by the JSS?	Free writing	
4	What is the position of the responder?	Multiple choice	Hospital Executive, Head of department, Staff, Other
5	What is the number of full-time surgeons per subspecialty employed at your institution?	Multiple choice	(Stratified by the sub-specialty) 0, 1, 2, 3, 4, 5, 6 or more
6	What is the number of surgical residents per subspecialty employed at your institution?	Multiple choice	(Stratified by the sub-specialty) 0, 1, 2, 3, 4, 5, 6 or more
7	Does your institution employ a part-time surgeon?	Multiple choice	Yes, No
8	What is the number of part-time surgeons per subspecialty employed at your institution?	Multiple choice	(Stratified by the sub-specialty) 0, 1, 2, 3, 4, 5, 6 or more
9	What is the percentage of anesthesia conducted by a surgeon for their own surgery?	Multiple choice	Always (≥ 90%), Sometimes (90%–10%), Rarely (< 10%)
10	What is the percentage of anesthesia conducted by a surgeon for surgery performed by other departments?	Multiple choice	Always (≥ 90%), Sometimes (90%-10%), Rarely (< 10%))
11	What is the percentage of chemotherapy administered to their own patients by the surgeons?	Multiple choice	Always (≥ 90%), Sometimes (90%–10%), Rarely (< 10%), Not applicable
12	How many days a week does a surgeon receive first calls from the emergency room during the day?	Multiple choice	Always (≥ 90%), Sometimes (90%–10%), Rarely (< 10%)
13	How many days a week does a surgeon receive first calls from the emergency room at night?	Multiple choice	Always (≥ 90%), Sometimes (90%–10%), Rarely (< 10%)
14	Please describe any duties that are burdensome for the surgeon	Free writing	
15	Do you feel there is a shortage of surgeons at your hospital?	Multiple choice	Yes No No definite answer
16	How many additional surgeons do you think your hospital needs?	Multiple choice	1–2 3–4 5 or more
17	How could the shortage of surgeons at your hospital be resolved?	Multiple choice	Increase in full-time surgeons Increase in part-time surgeons Increase in post-graduate residents or surgical residents Increase of staff in other departments Reduction of surgeon's total duties Reduction of duties other than operation Reduction of non-clinical duties Task shifting Others
18	How do you recruit the surgeons?	Multiple choice	Dispatch form university Private agency Surgical training system Hospital’s own effort Regional quota Others

### Definition of the main training facility and the partner training facility in the surgical training program by the JSS

Briefly, a main training facility must have at least 30 beds for the surgical department and perform at least 500 surgical procedures that are recorded in the National Clinical Database (NCD) [7]. A main training facility must also be registered as certified training facilities for at least three of the following four sub-specialties; gastroenterological surgery, cardiovascular surgery, general thoracic surgery and pediatric surgery, and at least three full-time board-certified surgeons must be employed. Conversely, registration of ≥ 50 surgical procedures in the NCD along with employment of one full-time board-certified surgeon is sufficient to be eligible as a partner training facility. A main training facility is expected to be responsible for a training program, in collaboration with at least one other hospital that plays the role of a partner training facility. A trainee must work as a full-time surgeon for at least 6 months both at the main training facility and at least one of the partner training facilities. A main training facility or a partner training facility of a training program can serve in the role of a partner training facility in one or more independent training programs, in which case a hospital will need to clarify how their patient resources are distributed to each of the training programs to which it belongs. A trainee is expected to participate as an operator or an assistant in at least 350 surgical procedures, which include a minimal number of surgeries from each of the following six subspecialties: gastroenterological surgery, cardiovascular surgery, general thoracic surgery, pediatric surgery, breast surgery and endocrine surgery. Each program is responsible for providing this compulsory surgical experience for all trainees within a training period; typically 3 years. For that purpose, the number of trainees that each program can accommodate is based strictly on the patient resources estimated from the recent track record of the participating hospitals. All the training programs undergo annual surveillance and need to be approved yearly by the JSS, and subsequently by the JMSB.

### Statistical analyses

Continuous data were compared using t-tests, and categorical data were compared using a chi-squared test. Logistic regression analysis was used for univariate and multivariate analyses. The cut-off values of the number of beds in the univariate and multivariate analyses at 210 beds and 804 beds were based on the receiver operating characteristic analysis. All statistical analyses were performed using JMP Pro version 16 1.0 (SAS Institute, Cary, NC, USA).

## Results

### Background and characteristics of the responding hospitals

The online survey was sent to all 1976 hospitals approved by the JSS and JMSB as either a main or a partner training facility, and responses were received from 1335 of these hospitals (67.6%). All the main training facilities, each of which oversees one of the 237 training programs, responded, as opposed to 1098 of the 1739 hospitals (63.1%) that were only partner training facilities. The responders were the director or vice-director of the hospital in 53.6%, a professor or head of the surgical department in 35.5%, another staff member involved in the teaching program in 8.7%, and others in 2.2%. The median number of beds in the responding hospitals was 313 (0–1,435). Table 2 summarizes the volume of the teaching hospitals. Most teaching hospitals employed a board-certified gastroenterological surgeon, whereas full-time certified surgeons in cardiovascular surgery, general thoracic surgery, pediatric surgery, breast surgery, and endocrine surgery were employed in 43.7%, 44.3%, 17.3%, 50%, and 9% of all teaching hospitals. As many as 879 (65.8%) hospitals employed one or more part-time surgeons. Table 3 summarizes the differences in various parameters between the main and partner training facilities.

**Table 2:** Background of the hospitals that responded to the survey

Role in the JSS surgical training program	
Main training facilities (response rate)	237 (100%)
Partner training facilities (response rate)	1,098 (63.1%)
Number of hospital beds	
19 beds or less	8 (0.6%)
20–300 beds	626 (46.9%)
301–600 beds	519 (38.9%)
601 beds or more	182 (13.6)
Board-certified surgeons of each of the 6 specialties as full-time hospital staff	
Gastroenterological surgery	1,231 (92.2%)
Cardiovascular surgery	584 (43.7%)
General thoracic Surgery	591 (44.3%)
Pediatric surgery	231 (17.3%)
Breast Surgery	668 (50.0%)
Endocrine Surgery	140 (9.0%)
Employment of more than one part-time surgeon	879 (65.8%)
Duties surgeons are assigned outside of their role as a surgeon	
Anesthesia for their own patients	276 (20.7%)
Anesthesia for other surgical treatments	113 (8.5%)
Chemotherapy for patients with cancer	1220 (91.4%)
First call in the emergency room during the day	771 (57.8%)
First call in the emergency room at night	1113 (83.4%)

**Table 3:** Comparison between the main training facilities and the partner training facilities

Variables	Main training facilities (n = 237)	Partner training facilities (n = 1,098)	P value
Median number of hospital beds (range)	647 (135–1435)	273 (0–1217)	< 0.0001
Number (%) of hospitals with full time surgeon in each specialty			
Gastroenterological surgery	236 (99.6%)	995 (90.6%)	< 0.0001
Cardiovascular surgery	233 (98.3%)	351 (32.0%)	< 0.0001
General thoracic Surgery	229 (96.6%)	362 (33.0%)	< 0.0001
Pediatric surgery	133 (56.1%)	98 (8.9%)	< 0.0001
Breast Surgery	219 (92.4%)	449 (40.9%)	< 0.0001
Endocrine Surgery	68 (28.7%)	72 (6.6%)	< 0.0001
Number (%) of hospitals that employ part-time surgeons	136 (57.4%)	743 (67.7%)	0.003
Duties surgeons are assigned outside their role as a surgeon			
Anesthesia for their own patients	26 (11.0%)	250 (22.8%)	< 0.0001
Anesthesia for other types of surgical treatment	3 (1.3%)	110 (10.0%)	< 0.0001
Chemotherapy for patients with cancer	220 (92.8%)	1000 (91.1%)	0.383
First call in the emergency room during the day time	71 (30.0%)	700 (63.8%)	< 0.0001
First call in the emergency room at night	159 (67.1%)	954 (83.4%)	< 0.0001
Number (%) of hospitals that suffer from a shortage of surgeons	121 (51.1%)	612 (55.7%)	0.347

### Shortage of surgeons

**Fig. 1:**
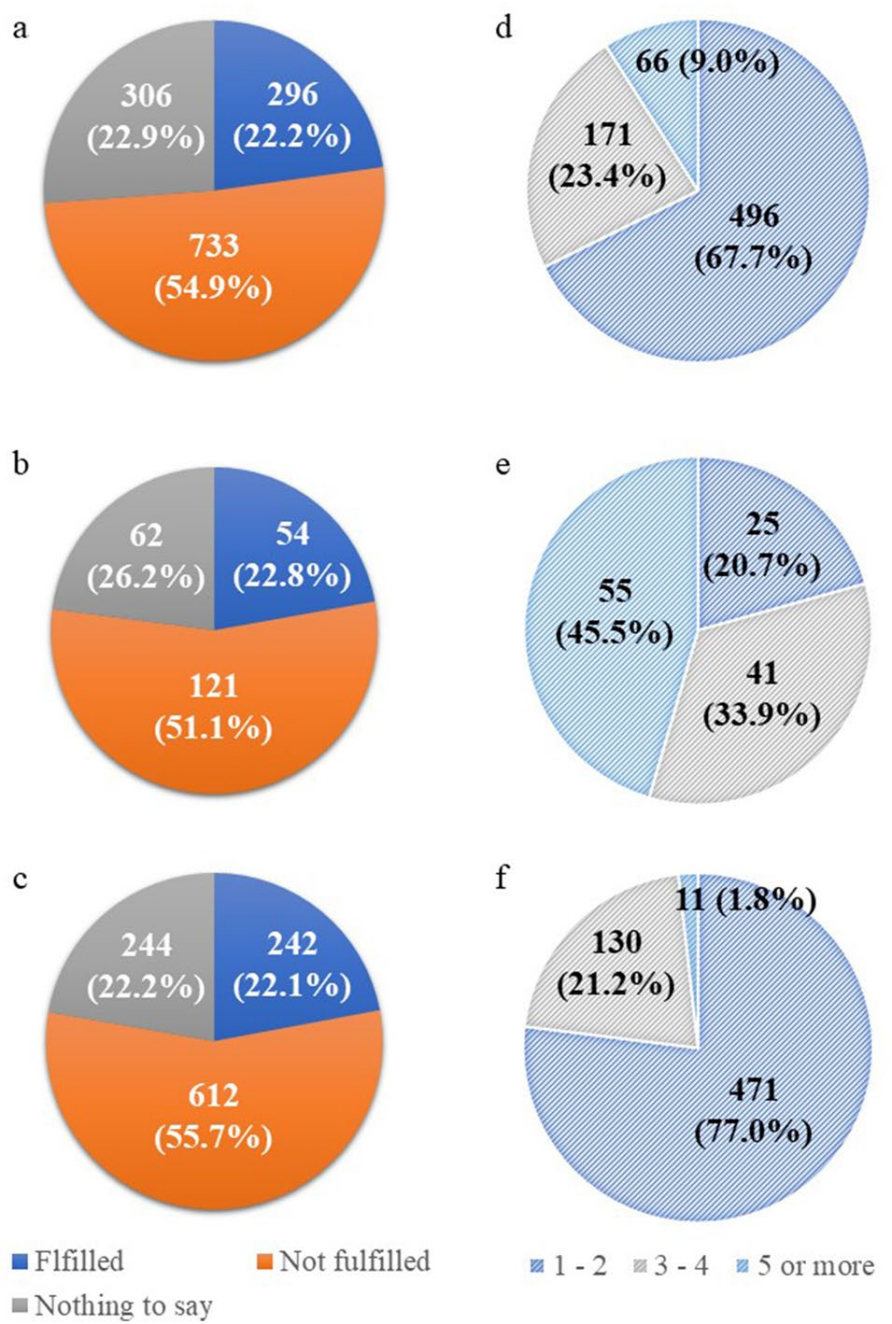
Shortage of surgeons. The figures on the left show responses to question 15 “Do you feel there is a shortage of surgeons at your hospital?” The figures on the right show responses to question 16 “How many additional surgeons do you think your hospital needs?” **a**, **d** depict responses from all teaching hospitals that responded,**b**, **e** depict responses from all 237 main training facilities, and**c**, **f** depict responses from 1098 of 1739 partner training facilities

**Fig. 2:**
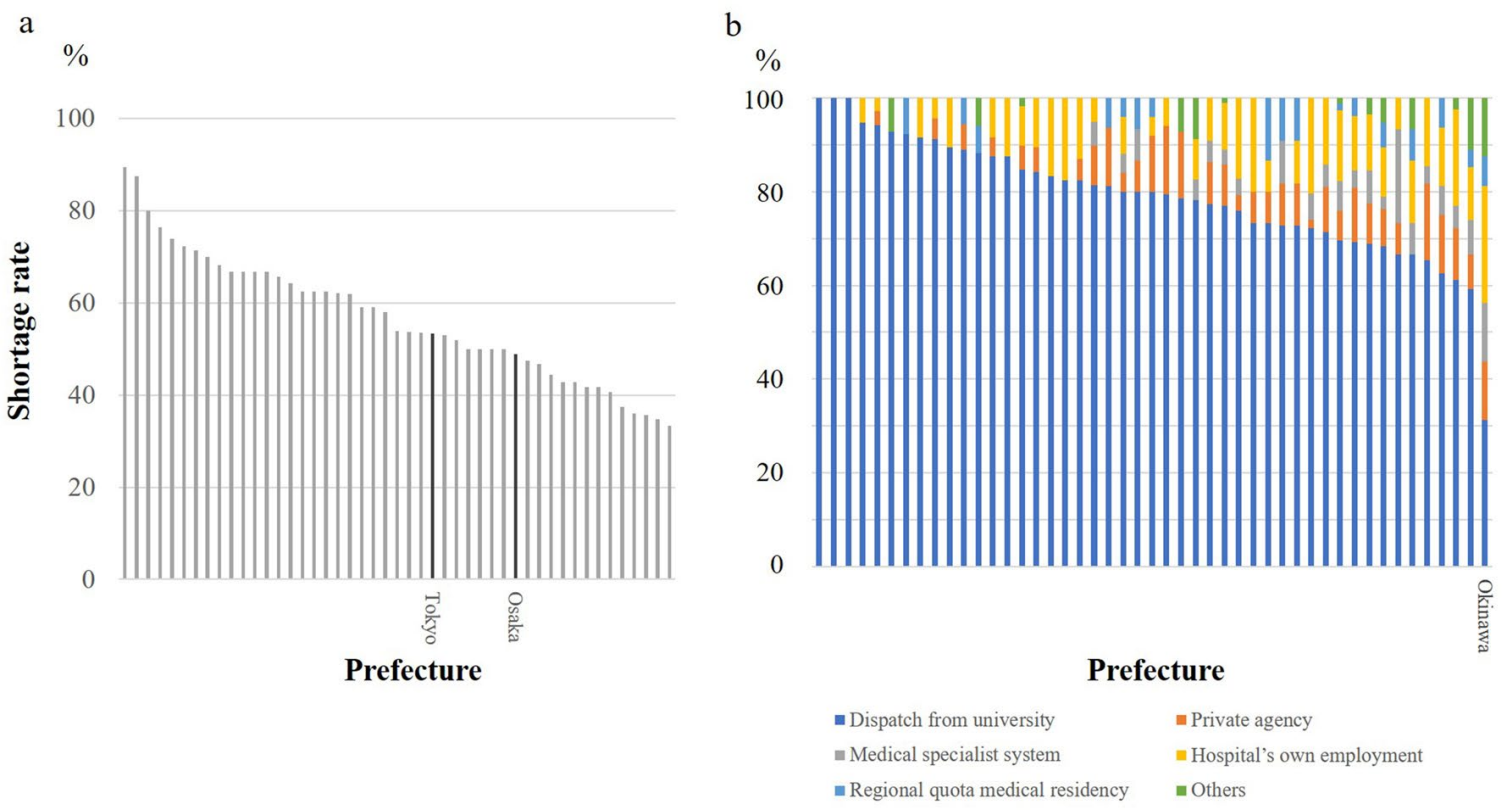
**a** The proportion of hospitals in each prefecture that answered “yes, definitely” to question 15 “Do you feel there is a shortage of surgeons at your hospital?” Only data from Tokyo and Osaka are labelled.**b**. Response to question 19 “How do you recruit surgeons?” stratified by the prefecture. Dispatch from university was the most frequent answer in all prefectures, with one exception

The number of surgeons was considered satisfactory in only 296 hospitals (22.2%), whereas responses from 733 hospitals (54.9%) indicated a shortage of surgeons (Fig. 1a). In response to the question of how many additional surgeons would be needed to run the surgical department adequately, 496 of the 733 hospitals (67.7%) reported needing 1–2 surgeons, 171 (23.4%) hospitals reported needing 3–4, and 66 (9.0%) hospitals reported needing 5 or more (Fig. 1d). Surprisingly, the shortage of surgeons was similar for the main training facilities, which are meant to have a leading position within a training program, and the partner training facilities (Fig. 1b, c). Reflecting the hospital volume, the number of additional surgeons needed tended to be greater for the main training facilities. In fact, nearly half of the main training facilities with a shortage of surgeons claimed that they needed five or more additional surgeons (Fig. 1e), whereas one additional surgeon was sufficient for 77% of the partner training facilities (Fig. 1f). Figure 2a shows the percentage of hospitals in each prefecture that declared a shortage of surgeons. The percentage ranged from under 40% to nearly 90%. While the maldistribution of physicians has been assumed to favor well-populated prefectures, in the current study, the percentages of hospitals that postulated a shortage of surgeons were not low in Tokyo and Osaka, which are two of the most populated prefectures in Japan.

### How the surgeons are recruited

Most hospitals (1046 of 1335; 78%) rely on the resources of a surgical department of the university hospital with which they have a close relationship (Fig. 2b). Generally, the university hospitals provide human resources for community hospitals, typically but not always, according to geographic proximity. While this traditional system can benefit the university hospitals from the viewpoint of securing staff positions for their graduates, it can impose a responsibility to support regional healthcare. Other methods of securing surgeons were through efforts made by the hospital, such as reputation as a good teaching hospital (124 hospitals; 9.3%); through private agencies that have a contract with surgeons not affiliated with a university (90 hospitals; 6.7%); from the main training facility of the surgical training program, being applicable only to the trainees of the new training system organized by the JMSB (43 hospitals; 3.2%); and through the system of regional reservation (25 hospitals; 1.8%). Okinawa was the only prefecture that did not rely excessively on the university hospital for the recruitment of surgeons (Fig. 2b).

Figure 2b depicts the methods used to recruit surgeons in each prefecture. Hospitals in most prefectures relied heavily on dispatch from the surgical departments of university hospitals. Private agencies supply a much smaller percentage of surgeons. Each prefecture reserves a fixed number of positions for medical students at a university every year as regional quota to train doctors who will spend at least a decade within the prefecture as a resident, trainee, and eventually a hospital doctor. However, this system does not seem to play a major role in the distribution of surgeons.

### Duties as anesthetists

**Fig. 3:**
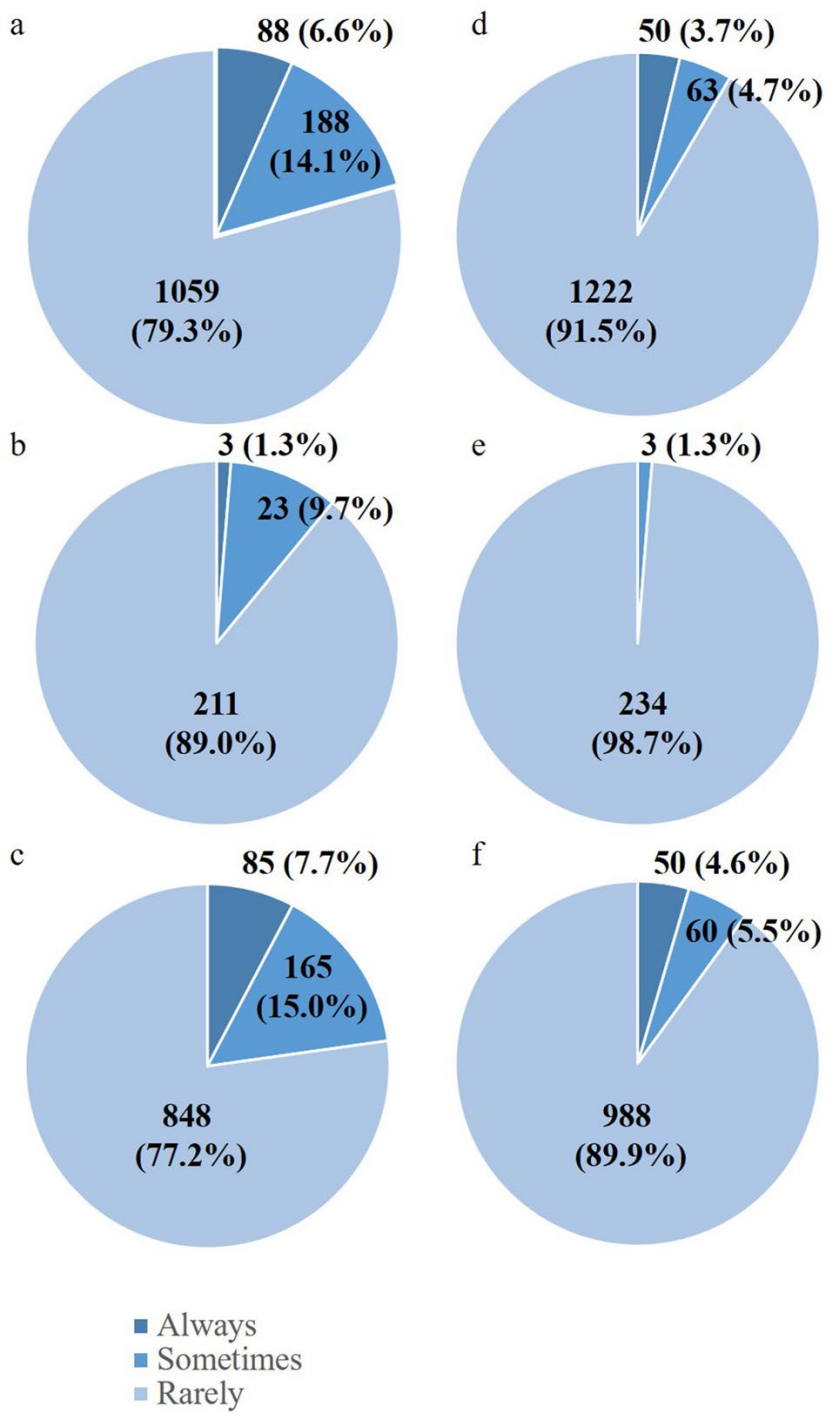
Duties in anesthesia. The figures on the left show responses to question 9 “What is the percentage of anesthesia conducted by a surgeon for their own surgery?” The figures on the right show responses to question 10 “What is the percentage of anesthesia conducted by a surgeon for surgery performed by other departments?”**a**, **d** depict responses from all teaching hospitals that responded,**b**, **e** depict responses from all 237 main training facilities and**c**, **f** depict responses from 1098 of 1739 partner training facilities. “Always”, “sometimes”, and “rarely” stand for > 90%, 10–90% and < 10%, respectively

As a result of the shortage of anesthetists and the paucity of certified registered nurse anesthetists, surgeons and surgical trainees are obliged to take on the role of anesthetists in some hospitals. Anesthesia for ≥ 90% of their own surgical procedures has been conducted by the surgeons themselves in 88 hospitals (6.6%), and for 10% ~ 90% of their own surgical procedures in an additional 188 hospitals (14.1%) (Fig. 3a). Surgeons were requested to serve as an anesthetist in ≥ 90% of procedures performed by other departments such as urology, otolaryngology, and ophthalmology, in addition to their own surgery in 50 hospitals (3.7%), and in 10% ~ 90% of all surgery in an additional 63 hospitals (4.7%) (Fig. 3d). In the main training facilities, the number of surgical departments that anesthetize ≥ 90% and 10% ~ 90% of their own patients declined to 3 (1.3%) and 23 (9.7%), respectively (Fig. 3b), and the number and percentage of surgical departments that anesthetize ≥ 90% and 10% ~ 90% of all surgical procedures decreased to 0 and 3 (1.3%) (Fig. 3e). Conversely, the number and percentage of surgical departments that anesthetize ≥ 90% and 10% ~ 90% of their own patients was as high as 85 (7.7%) and 165 (15.0%), respectively in the partner training facilities (Fig. 3c), and the number and percentage of surgical departments that anesthetize ≥ 90% and 10% ~ 90% of all surgical procedures rose to 50 (4.6%) and 60 (5.5%).

### Duties as medical oncologists

**Fig. 4:**
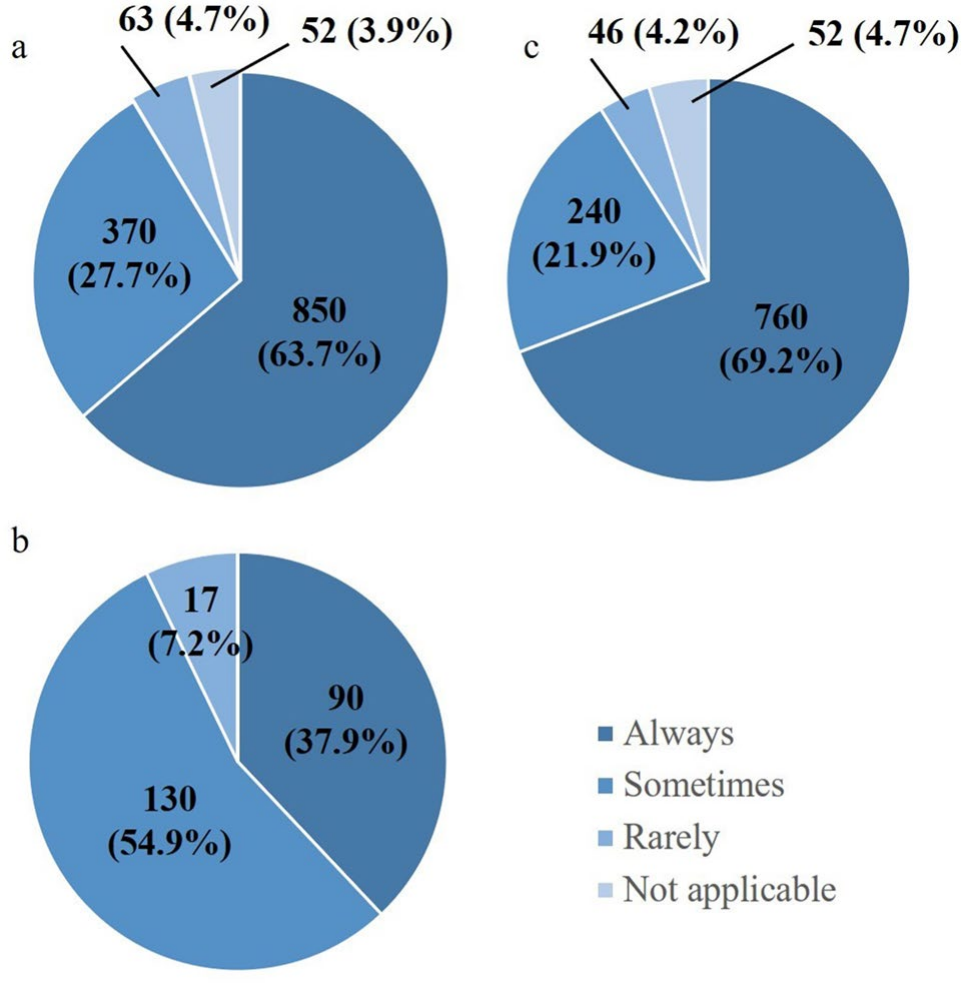
Duties in medical oncology. The figures show response to question 11 “What is the percentage of chemotherapy administered to their own patients by the surgeons?” **a** depicts responses from all teaching hospitals that responded, **b** depicts responses from all 237 main training facilities, and **c** depicts responses from 1098 of 1739 partner training facilities. “Always”, “sometimes”, and “rarely” stand for > 90%, 10–90% and < 10%, respectively

In line with the focus on early diagnosis, which has improved the outcome of gastric cancer in particular, most physicians who major in gastroenterology are essentially endoscopists who continue to devote their time and effort to diagnosis and endoscopic resection or intervention. Their interest in the field of basic research is focused on carcinogenesis. Consequently, there remains a serious shortage in the number of medical oncologists, particularly in the field of gastroenterology, and the number and percentage of hospitals in which a surgeon delivers chemotherapy to ≥ 90%, 10% ~ 90%, and 0% ~ 10% of patients with advanced or recurrent cancer are 850 (64%), 370 (28%), and 63 (8.6%), respectively (Fig. 4a). The percentage of hospitals where surgeons deliver chemotherapy to ≥ 90%, 10% ~ 90%, and 0% ~ 10% of patients was 38%, 55%, and 7% in the main training facilities (Fig. 4b) and 69%, 22%, and 9% in the partner training facilities (Fig. 4c). This denotes that surgeons take on the role of medical oncologists far more often than they take on the role of anesthetists in both main and partner training facilities.

### Duties in the emergency room

**Fig. 5:**
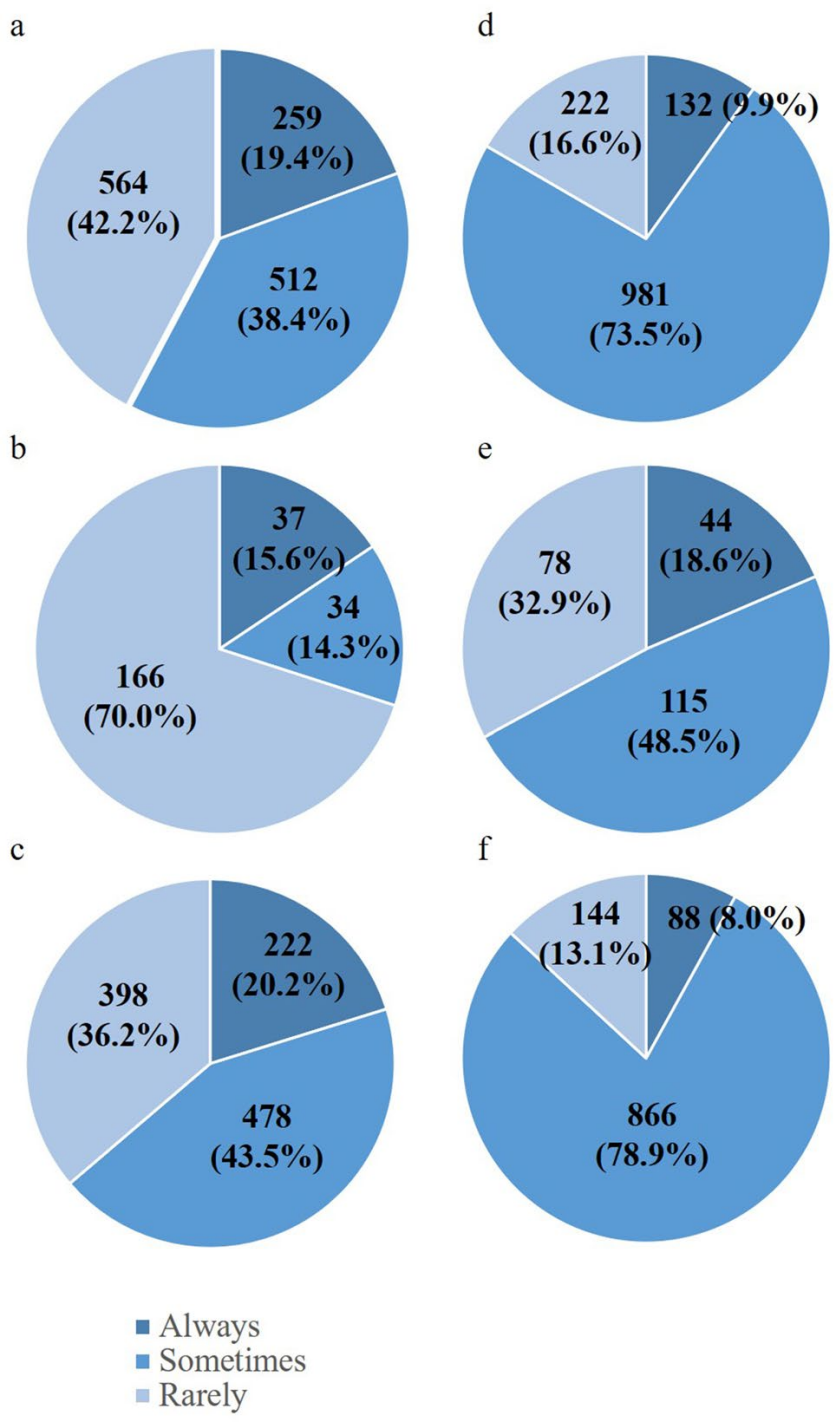
Duties in the emergency room. The figures on the left show responses to question 12 “How many days a week does a surgeon receive first calls from the emergency room during the day?” The figures on the right show response to question 13 “How many days a week does a surgeon receive first calls from the emergency room at night?” **a**, **d** depict responses from all teaching hospitals that responded,**b**, **e** depict responses from all 237 main training facilities, and **c**, **f** depict responses from 1098 of 1739 partner training facilities. “Always”, “sometimes”, and “rarely” stand for “almost every day”, “a few days a week” and “rarely”, respectively

The absence of emergency physicians in many hospitals has resulted in surgeons often taking the first call from the emergency room when patients present with injury or disease requiring surgery. Figure 5 depicts how often the first-call role is assigned to a surgeon during the day (Fig. 5a) and night (Fig, 5d). The data were then stratified into the main (Fig. 5b, e) and partner (Fig. 5c, f) training facilities. It is clear that most partner training facilities rely on a surgeon for the first-call duties, especially at night.

### The difference in various parameters between the main training facilities and partner training facilities (Table 3*)*

A main training facility has greater number of beds and is more likely to be staffed by surgeons with specialties in addition to the gastroenterology. Pediatric and endocrine surgeons are not always employed, even in the main training facilities, and patients in these disciplines are likely to be centralized to specific hospitals. Although surgeons at partner training facilities are more often involved in duties as an anesthetist and in the emergency room, surgeons in hospitals in both categories are invariably burdened with delivering chemotherapy, which is inevitably accompanied by end-of-life care.

### Predictive factors of surgeon shortage at teaching hospitals

The burden of delivering chemotherapy to patients with advanced recurrent cancer (odds ratio 1.473: 95% CI 1.004 ~ 2.162, p = 0.048), working in an emergency room during the day (odds ratio 1.276: 95% CI 1.026 ~ 1.587, p = 0.029), and at night (odds ratio 1.412: 95% CI 1.058 ~ 1.884, p = 0.019) were identified as significant predictors of the shortage of surgeons at all teaching hospitals by univariate analysis (Table 4). A hospital with ≥ 210 beds (odds ratio 1.443: 95% CI 1.118 ~ 1.862, p = 0.003) and the role of a surgeon as an anesthetist for surgical procedures in other departments in addition to their own surgery (odds ratio 1.643: 95% CI 1.074 ~ 2.512, p = 0.022) were also identified as independent predictors of the shortage in surgeons (Table 4). An independent predictor of the shortage of surgeons at main training facilities was the number of beds at ≥ 804 (odds ratio 2.151, 95% CI 1.172 ~ 3.949, p = 0.013) (Table 5). Independent predictors of the shortage of surgeons at partner training facilities were the number of beds at ≥ 210 (odds ratio 1.534, 95% CI 1.176 ~ 2.001, p = 0.002), and the burden of anesthesia responsibilities in other departments in addition to their own patients (OR 1.705, 95% CI 1.104 ~ 2.635, p = 0.016) (Table 6). Variance inflation factors for all variables included in the multivariate analyses were confirmed to be less than 5.

**Table 4:** Predictors of a shortage surgeons in all teaching hospitals: Univariate and multivariable analyses

Variables	Univariate			Multivariable		
	Odds ratio	95% CI	P** value**	Odds ratio	95% CI	P value
Bed number (≥ 210 beds)	1.242	0.990–1.583	0.061	1.443	1.118–1.862	0.003
The principal hospital	0.828	0.625–1.097	0.189			
Anesthesia in the surgical department	1.106	0.847–1.445	0.460			
Anesthesia in non-surgical departments	1.490	0.999–2.223	0.050	1.643	1.074–2.512	0.022
Chemotherapy	1.473	1.004–2.162	0.048	1.332	0.901–1.970	0.151
The first call for emergency patients during the day	1.276	1.026–1.587	0.029	1.238	0.975–1.572	0.080
The first call for emergency patients during the night	1.412	1.058–1.884	0.019	1.269	0.935–1.721	0.127

**Table 5:** Predictors of a shortage of surgeons at the main training facilities: Univariate and multivariable analyses

Variables	Univariate			Multivariable		
	Odds ratio	95% CI	P value	Odds ratio	95% CI	P value
Bed number (≥ 804 beds)	2.151	1.172–3.949	0.013	2.151	1.172 – 3.949	0.013
Anesthesia in the surgical department	0.673	0.295–1.534	0.347			
Anesthesia in the non-surgical department	0.475	0.042–5.311	0.546			
Chemotherapy	0.922	0.343–2.477	0.872			
The first call for emergency patients during the day	1.062	0.609–1.853	0.831			
The first call for emergency patients during the night	1.241	0.721–2.135	0.436

**Table 6:** Predictors of a shortage of surgeons at the partner training facilities: Univariate and multivariable analyses

Variables	Univariate			Multivariable		
	Odds ratio	95% CI	P value	Odds ratio	95% CI	P value
Bed number (≥ 210 beds)	1.362	1.063–1.744	0.014	1.534	1.176–2.001	0.002
Anesthesia in the surgical department	1.151	0.865–1.531	0.334			
Anesthesia in the non-surgical department	1.504	0.998–2.267	0.051	1.705	1.104–2.635	0.016
Chemotherapy	1.614	1.063–2.449	0.025	1.442	0.940–2.211	0.093
The first call for emergency patients during the day	1.287	1.005–1.648	0.046	1.259	0.967–1.639	0.087
The first call for emergency patients during the night	1.436	1.011–2.040	0.043	1.244	0.863–1.795	0.242

### Thoughts of the hospital authorities on how to resolve the shortage of surgeons

**Fig. 6:**
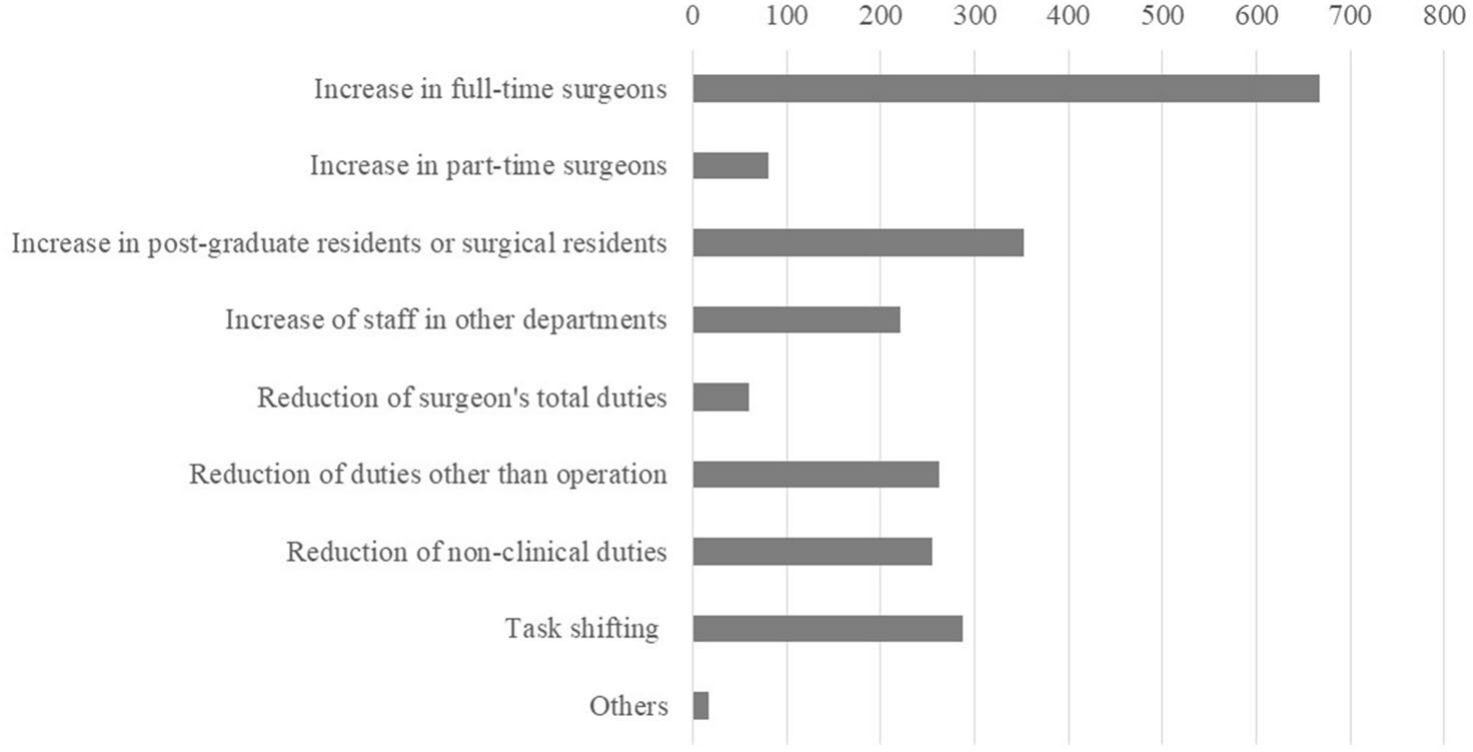
Responses to question 16 “How could the shortage of surgeons at your hospital be resolved?” are shown. The responders were allowed to choose more than one from the list of alternatives

Figure 6 summarizes the responses to question No. 17. The most frequently chosen solution was to increase the number of full-time surgeons, followed by attempts to increase the number of post-graduate residents or surgical residents. Conversely, engaging part-time surgeons was not considered helpful. While the hospital authorities are counting on task shifting which involves contributions from hospital workers other than the doctors, fundamental remedies may require the engagement of specialists such as medical oncologists, anesthesiologists, and acute care physicians, who can allow surgeons to focus on their surgical responsibilities.

## Discussion

Upon the establishment of a new system of board certification for 19 fundamental specialty areas including surgery, the JMSB urged societies of the core specialties to create a sufficient number of training programs. It was stipulated that each program must be run by one main training facility with the assistance of more than one partner training facility. Moreover, the program should not involve only high-volume hospitals from large cities, but include hospitals from rural areas as partner training facilities. During the 3 years of training, trainees must work for at least 6 months at both the main training facility and one of the partner training facilities. The aim of the JMSB, apparently reflecting the intention of the Ministry of Health, Labor, and Welfare, was that a proportion of trainees will consequently work for partner training facilities in the rural areas. The JMSB went as far as to establish a rule by which the doctors should be appointed to work for 1 year at the hospitals in the region that suffer from a shortage of physicians, within 3 years after acquiring board certification. All societies of the 19 fundamental specialty areas opposed this rule, which apparently aims to solve the issue of maldistribution of doctors by making inappropriate use of the board certification system. The JMSB eventually compromised and turned the “rule” into a “recommendation”. Nevertheless, the JSS sought to identify the current status on the distribution of surgeons to prepare for future attempts to resolve the issue independently rather than through forceful intervention by the government. This is why the current survey was conducted.

Traditionally, the clinical departments of each university have been associated with community hospitals, typically in their region, but sometimes in other parts of Japan [8]. Having a large number of associated hospitals would mean that the members of a clinical department could find a position within a wide range of hospitals when they complete their doctoral thesis and leave the department. On the other hand, the department would be responsible for the well-being of all their associated hospitals and consequently, of the regional healthcare. In this way, a hospital owes much to the effort of the university clinical department with which it has a close relationship, to secure enough doctors, whereas a clinical department will need to support all associated hospitals by placing their doctors, sometimes disregarding the wish of individual doctor. In other words, the university clinical departments work as an internal labor market central to the careers and professional lives of physicians [8]. Although the government has been reluctant to acknowledge the fact that regional healthcare is dependent on the university hospitals, it is indisputably evident from the current survey that the university surgical departments play a dominant role in supporting the community hospitals through dispatching surgeons, no matter whether one considers it as a contribution to the regional healthcare or a dictatorship. There was only one prefecture where the role of university surgical department was not dominant regarding the recruitment of surgeons by the regional hospitals.

Unfortunately, fewer than a quarter of all teaching hospitals answered that they are staffed with a sufficient number of surgeons, whereas over 50% of hospitals claimed that they are understaffed. Such dismal responses were evident throughout the country, even in prefectures with large cities such as Tokyo and Osaka (Fig. 1a). The proportion of hospitals with a shortage of surgeons was similar across the two categories of hospitals, with major training facilities often needing more than three surgeons. In fact, hospitals with the number of beds exceeding the cutoff value was a predictor of surgeon shortage in both major training facilities and partner training facilities, suggesting that larger hospitals are apt to be burdened with greater workload and little extra support. A lower incidence of surgical mortality and failure to rescue in high-volume hospitals is well documented across several types of surgery [9, 10]. Furthermore, diseases that require complex surgery tend to be centralized. According to the big data from the NCD, the involvement of board-certified surgeons at high-volume hospitals in Japan led to a prominent decline in 90-day mortality rates for complex surgery such as esophagectomy, pancreatico-duodenectomy, and hepatectomy [11]. Thus, large-scale hospitals such as those that take on the role of main training facilities are consistently in need of larger surgical teams capable of covering a wider range of specialties. These viewpoints and the disclosure by the Ministry of Health, Labor, and Welfare at the JMSB meetings that the total number of surgeons remains insufficient (data not shown), show that merely redistributing surgeons from urban district to rural areas or from high-volume hospitals to small-scale hospitals is not the solution to overcoming the shortage of surgeons in Japan.

It is intriguing that the number of surgeons per 100,000 inhabitants is reported to be larger in Japan than in the US [12]. The shortage of surgeons despite these data can be explained by the diverse roles of surgeons in Japan, which are not common in other developed countries. Besides taking on important roles in the emergency room, many surgeons take on the role of medical oncologists to manage patients with unresectable or recurrent cancer. Although not specifically asked in this survey, these surgeon/oncologists are likely to be looking after their patients until death. End-of-life care is an extremely important part of cancer management but very different from the clinical activities that surgeons are trained for. The current shortage of medical oncologists was identified by univariate analyses as a potential burden to surgeons, as was taking on the role of anesthetists, especially at the partner training facilities. A department of anesthesiology seems to be non-existent in 8.4% of all teaching hospitals where surgeons are asked to be almost completely responsible for this role. Even when anesthetists are available, surgeons are still asked to serve as anesthetists for their own surgery to cover the shortage of anesthetists in 20% of all hospitals. This situation should be amended, especially given the fact that the number of anesthetists is increasing remarkably in Japan, in stark contrast to the declining number of surgeons.

The ultimate solution is to increase the number of surgical residents; however, the number of trainees who join the surgical training program has not increased in Japan despite the recent increase in the number of graduates through expansion of capacity at medical schools. This is because surgery is a demanding career with great rewards but with equally great physical, psychological, and spiritual challenges [5]. Efforts must be made to address the rigorous lifestyle of surgeons and to adjust for the physician’s work style reforms, which will be activated in April, 2024 [6]. Forceful recruitment in the absence of improvements in the well-being of surgeons will only lead to attrition from training programs. The attrition rate averages 18% [4], even in the US where being a surgeon seems to be economically more rewarded, and a significant gender difference in the attrition rate still exists in Canada where efforts for gender equity have been in action for much longer than in Japan [13]. Perhaps it is time to appreciate the fact that many residents in their initial residency program still wish to join the surgical training program each year in Japan. Their professional life would become more rewarding if they could concentrate on surgical training rather than being obliged to give their time to other miscellaneous duties. Historically, surgeons have been competent and well-trained in activities of clinical practice that are essentially outside of their true expertise and have voluntarily covered various departments missing in each hospital. The time has come to seriously consider protecting surgeons from being excessively distracted from their true job as surgeons/surgical trainees so that well-trained surgeons will be available in all community hospitals where surgical treatments need to be offered.

This study has several limitations. The response rate of the partner training facilities was low, at 63.1%, whereas all the main training facilities responded. Since the number of surgical trainees in Japan at around 800 per a year is much smaller than the capacity of the whole training system at > 2000, there were several partner training facilities without trainees and these hospitals, which are apt to be smaller in scale and could be situated in more rural areas, may have been the ones that did not respond. Thus, the responses from the partner training facilities may have been biased. The response rates according to the prefecture was also varied, ranging from 86% in Saga prefecture to 46% in Tottori prefecture. We decided not to disclose the information about which data came from which prefecture to avoid comparisons that could be potentially misleading, with the exception of data from Tokyo (response rate: 74%) and Osaka (response rate: 70%). In question 19, it was unclear whether a surgeon to be recruited was a qualified surgeon or confined to surgical trainees. One of the alternatives that could be selected from the multiple choice was that a surgeon could be recruited through the surgical training system, but that applies only to the surgical trainees.

In conclusion, the current shortage of surgeons is a serious issue throughout Japan, including in well-populated prefectures such as Tokyo and Osaka. The departments of surgery at university hospitals remain a major source of surgeons across most prefectures. Importantly, responsibilities in fields such as anesthesiology and medical oncology are a potential burden to surgeons. Given the limited number of surgeons and surgical trainees, hospital authorities should make every effort to recruit professionals in these fields and allow surgeons to engage more in surgery.

### Supplementary Information

Below is the link to the electronic supplementary material.

Supplementary file1 (DOCX 13 kb)

## Data Availability

The participants of this survey did not give written consent for their data to be shared publicly. Due to the sensitive nature of the research, supporting data is not generally available.
