# Supplementary material for: The shortage of surgeons in Japan: Results of an online survey of qualified teaching hospitals that take part in the surgical training programs for board certification by the Japan Surgical Society
Source: Surg Today. 2023 May 16;54(1):41–52. doi: 10.1007/s00595-023-02697-7 (PMC10764368; doi:10.1007/s00595-023-02697-7)
Supplement: Supplementary file 1 — Supplementary file1 (DOCX 13 kb) [file 595_2023_2697_MOESM1_ESM.docx]

| Number of full-time surgeons per subspecialty | ≥6 | 5 | 4 | 3 | 2 | 1 | 0 |
| --- | --- | --- | --- | --- | --- | --- | --- |
| Gastroenterological surgery | 443 | 138 | 179 | 190 | 170 | 111 | 104 |
| Cardiovascular surgery | 104 | 60 | 67 | 115 | 111 | 127 | 751 |
| Respiratory Surgery | 51 | 25 | 48 | 91 | 174 | 202 | 744 |
| Pediatric surgery | 20 | 8 | 25 | 40 | 48 | 90 | 1104 |
| Breast Surgery | 43 | 12 | 38 | 68 | 184 | 323 | 667 |
| Endocrine Surgery | 10 | 4 | 10 | 10 | 34 | 72 | 1195 |
| Acute care Surgery | 78 | 12 | 15 | 25 | 64 | 131 | 1010 |
| Others | 16 | 7 | 19 | 23 | 70 | 101 | 1099 |

Supplemental Table 1
